# Centimeter-scale Green Integration of Layer-by-Layer 2D TMD vdW Heterostructures on Arbitrary Substrates by Water-Assisted Layer Transfer

**DOI:** 10.1038/s41598-018-37219-w

**Published:** 2019-02-07

**Authors:** Jung Han Kim, Tae-Jun Ko, Emmanuel Okogbue, Sang Sub Han, Mashiyat Sumaiya Shawkat, Md Golam Kaium, Kyu Hwan Oh, Hee-Suk Chung, Yeonwoong Jung

**Affiliations:** 1 NanoScience Technology Center, University of Central Florida, Orlando, Florida 32826 USA; 2 Department of Electrical and Computer Engineering, University of Central Florida, Orlando, Florida 32816 USA; 3 Department of Material Science and Engineering, Seoul National University, Seoul, 08826 South Korea; 4 Analytical Research Division, Korea Basic Science Institute, Jeonju, 54907 South Korea; 5 Department of Materials Science and Engineering, University of Central Florida, Orlando, Florida 32826 USA

## Abstract

Two-dimensional (2D) transition metal dichalcogenide (2D TMD) layers present an unusually ideal combination of excellent opto-electrical properties and mechanical tolerance projecting high promise for a wide range of emerging applications, particularly in flexible and stretchable devices. The prerequisite for realizing such opportunities is to reliably integrate large-area 2D TMDs of well-defined dimensions on mechanically pliable materials with targeted functionalities by transferring them from rigid growth substrates. Conventional approaches to overcome this challenge have been limited as they often suffer from the non-scalable integration of 2D TMDs whose structural and chemical integrity are altered through toxic chemicals-involved processes. Herein, we report a generic and reliable strategy to achieve the layer-by-layer integration of large-area 2D TMDs and their heterostructure variations onto a variety of unconventional substrates. This new 2D layer integration method employs water only without involving any other chemicals, thus renders distinguishable advantages over conventional approaches in terms of material property preservation and integration size scalability. We have demonstrated the generality of this method by integrating a variety of 2D TMDs and their heterogeneously-assembled vertical layers on exotic substrates such as plastics and papers. Moreover, we have verified its technological versatility by demonstrating centimeter-scale 2D TMDs-based flexible photodetectors and pressure sensors which are difficult to fabricate with conventional approaches. Fundamental principles for the water-assisted spontaneous separation of 2D TMD layers are also discussed.

## Introduction

In pursuit of ever-increasing technological developments in modern electronics, there exists a continued quest for exploring novel electronic materials which can outperform traditional thin film semiconductors as well as offer unprecedented functionalities absent in them. In this regard, two-dimensional (2D) transition metal dichalcogenide (2D TMD) atomic layers have recently gained enormous interests owing to their near ideal combination of superior electrical and mechanical properties unattainable in any conventional materials^1–10^. For instance, they can tolerate significant mechanical deformation owing to unusually high in-plane strain limits - i.e. >5 times over covalently-bonded inorganic thin films - while preserving intrinsic semiconductor properties manifested by large ON/OFF current ratios demanded for modern transistors^11,12^. Such property advantages project unexplored opportunities in a wide range of emerging technologies, particularly in digital electronics of unconventional form factors such as wearable and stretchable devices. Moreover, heterogeneously integrating 2D TMD layers of distinguishable yet tailored components has been predicted to enable even more exotic functionalities impossible with conventional thin film semiconductor growth technologies^3,13–25^. As 2D TMD layers exert weak van der Waals (vdW) attraction to underlying growth substrates, it is possible to individually assemble them in a layer-by-layer manner achieving targeted electronic structures, implying new venues for 2D heterojunction devices with tailored band offsets^22–26^. Such atom thick semiconductor heterostructures have been technically challenging to integrate with conventional thin film growth strategies owing to their intrinsic lattice match constraint which imposes the crystallographic limitation for the choice of materials to be integrated. A few critical prerequisites exist in order to realize the aforementioned advantages inherent to 2D TMDs toward their exploration for novel technologies; (1) It is demanded to develop viable strategies to transfer 2D TMD layers from original growth substrates and integrate them on secondary substrates of targeted functionalities, e.g., mechanically flexible substrates. (2) The intrinsic mechanical and electrical properties of the transferred 2D layers should not be compromised throughout their integration process and should be uniformly preserved on a wafer scale. (3) The layer integration process should be generalized to 2D TMDs and substrates of diverse materials without being limited to specific kinds for technological versatility. Presently, the most commonly employed approach for the transfer and integration of 2D TMD layers relies on the chemical etching of underlying growth substrates involving protection polymers (e.g., polymethyl-methacrylate (PMMA)) and subsequent chemical lift-off^27–29^. However, such strategies tend to result in the fragmentation of individual 2D layers as they employ solution-based chemicals to etch away both the protection layer and growth substrates (e.g., silicon dioxide (SiO_2_) or sapphire wafer)^28^. Accordingly, they impose scalability limitation in terms of heterogeneously stacking up 2D layers of multiple components in a controlled manner as well as being difficult to be applied to a variety of unconventional substrates. Moreover, the intrinsic material properties of 2D layers are often compromised and damaged by the employed chemicals though their transfer and integration stages.

Herein, we report a generic and reliable strategy to achieve the layer-by-layer integration of 2D TMDs of controlled morphology and component onto arbitrary substrates over a large area. The newly developed 2D layer integration method is intrinsically “green” as it employs water only without involving any other chemicals, thus is free of any chemicals-associated material degradation. We demonstrate the layer-by-layer integration of centimeter-scale (>2 cm^2^) uniform 2D TMDs and their heterostructures onto virtually arbitrary substrates including, papers, woods, and plastics, which is difficult to achieve with any conventional approaches. The integrated 2D TMD layers and their heterostructures well preserve their original structural and compositional integrity benefiting from the intrinsic cleanness of the water-assisted process, confirmed by extensive spectroscopy and microscopy characterization. The technological versatility of this 2D layer integration method has been demonstrated by developing large-area 2D TMDs-based flexible photodetectors and pressure sensors on plastics and papers, respectively.

## Results

### Procedure for water-assisted 2D layer integration

**Figure 1:**
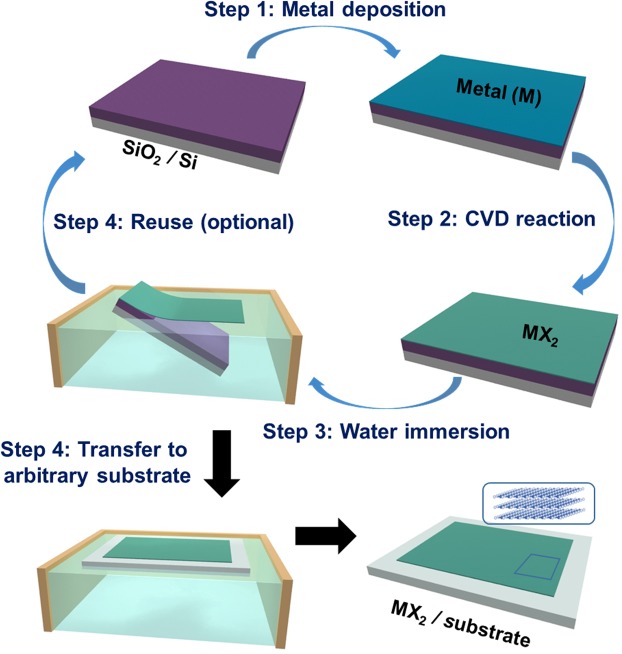
Schematic to illustrate the water-assisted green integration of CVD-grown 2D TMD layers on arbitrary substrates.

Figure 1 schematically depicts the integration of large-area 2D TMDs onto a variety of substrates via the water-assisted 2D layer transfer. The process is carried out in following steps: (1) Deposition of transition metals on the surface of growth substrates (i.e., SiO_2_/Si) followed by their conversion to 2D TMD layers via chemical vapor deposition (CVD). (2) Immersion of the 2D TMDs-grown SiO_2_/Si substrates inside water followed by spontaneous 2D layer separation. (3) Transfer and integration of the delaminated 2D TMD layers onto secondary substrates inside water. (4) Recycling of the original growth substrates for additional 2D TMDs growth (optional). Details for the CVD growth of 2D TMDs are described in Methods section. The water-assisted 2D layer separation can be carried out in following two slightly different manners. The first method is to slowly immerse the entire 2D TMDs-grown substrate inside water (See the video in Supplementary Information, S1) while the second method is to deposit water droplet only on the sample surface to leverage its buoyancy (See the video in Supplementary Information, S2). We emphasize that the whole process utilizes water only without involving any kinds of additional chemicals for the separation, transfer, and integration of 2D TMD layers. Previous approaches generally employed the combined use of polymeric protective materials (e.g., PMMA or Polyvinyl alcohol (PVA)) and chemical etchants for the removal of SiO_2_^28,29^. In addition to the operational complexity inherent to these conventional approaches, 2D TMDs are susceptible to chemical degradation owing to the corrosive nature of hydrogen fluoride (HF) or strong bases (sodium or potassium hydroxide (NaOH or KOH)) involved in the processes^27,29^. Moreover, their structural integrity can be significantly altered by the solution-based chemicals (e.g. acetone) employed to rinse away the protective polymers as well as being affected by the polymer residuals. On the other hand, our water-assisted approach yields the completely clean and homogenous integration of a variety of 2D TMDs on a large centimeter scale (to be demonstrated below). As this approach is intrinsically free of introducing chemicals-associated structural damage, the original SiO_2_/Si substrates after 2D layer separation can be reused for the subsequent growth of additional 2D TMD materials (step (4) in Fig. 1).

### Demonstration of water-assisted 2D layer transfer and integration

**Figure 2:**
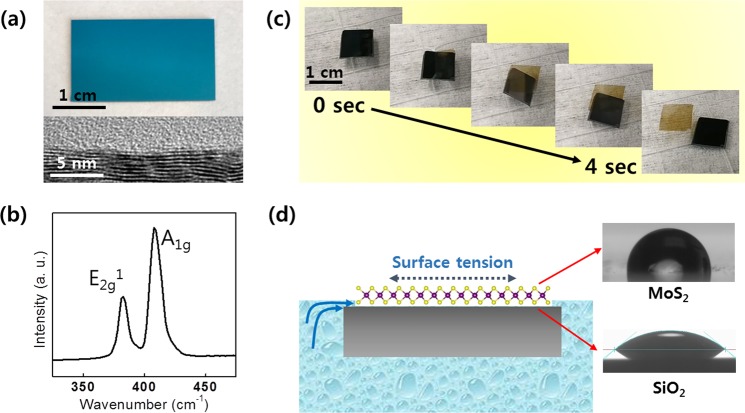
(**a**) Image of 2D MoS_2_ layers as-grown on SiO_2_/Si and the corresponding cross-sectional HRTEM image. (**b**) Raman spectrum obtained from the 2D MoS_2_ layers denoting their characteristic peaks. (**c**) Time-lapsed snapshot images to demonstrate the water-assisted spontaneous separation of 2D MoS_2_ layers inside water. (**d**) Illustration of the water penetration process at the 2D MoS_2_/SiO_2_ interface along with the representative images of water contact angle measurements for 2D MoS_2_ layers and SiO_2_.

Figure 2 is the experimental demonstration of the water-assisted 2D TMD layer transfer and integration utilizing 2D molybdenum disulfide (2D MoS_2_) as a representative case. Figure 2(a) shows the representative images of as-grown 2D MoS_2_ layers on SiO_2_/Si on a dimension of ~ 2 × 3 cm^2^ (top image). In optimizing 2D MoS_2_ layers growth conditions, we have considered the previous studies which report the transition of horizontal-to-vertical 2D layer orientation in the CVD thermal sulfurization of Mo^30–32^. In order to ensure the growth of horizontally-oriented layers only, we have typically deposited Mo of small and uniform thickness (<3 nm) and carried out the CVD sulfurization using the previously developed recipe^33–35^. The cross-sectional transmission electron microscope (TEM) characterization (bottom image) of the same sample represents well resolved, all horizontally-oriented 2D MoS_2_ layers. Figure 2(b) shows the Raman spectroscopy characterization of the same sample, revealing two distinguishable peaks corresponding to their in-plane and out-of-plane E^1^_2g_ and A_1g_ oscillation modes^36,37^. Figure 2(c) shows the time-lapsed representative images to demonstrate the sequential delamination of 2D MoS_2_ layers inside water recorded for ~4 sec (corresponding to the video in Supplementary Information, S2). As shown in the images, 2D MoS_2_ layers become spontaneously separated from the underlying SiO_2_/Si substrate and subsequently float on the water surface maintaining their original size and shape. The success of this facile water-assisted delamination of 2D layers is attributed to the surface energy imbalance between 2D MoS_2_ basal planes and SiO_2_ surfaces, as depicted in Fig. 2(d). Upon exposure to water droplet, our CVD-grown 2D MoS_2_ layers exhibit significantly larger hydrophobicity compared to the underlying SiO_2_, as demonstrated by the water contact angle measurement results. The observation is consistent with the previous studies on the surface wettability of 2D MoS_2_ layers exposing their basal planes for water adsorption^38–40^. Accordingly, upon the onset of water penetration into any opened interfaces of 2D MoS_2_/SiO_2_, mechanical tension is to occur and propagate underneath the weakly vdW-bound 2D basal planes. In other words, the distinct surface wettability of 2D MoS_2_ vs. SiO_2_ indicates a strong tendency for water repulsion from 2D MoS_2_ vs. water attraction to SiO_2_, respectively^27,41,42^. Moreover, it has been well known that the surface energy of 2D MoS_2_ layers is also sensitive to air exposure and its duration period. 2D MoS_2_ layers exhibit more pronounced hydrophobicity after elongated air exposure, confirmed by the water contact angle measurements in various studies^40,43,44^. In fact, we note that our CVD-grown 2D MoS_2_ layers also tend to become more easily separable inside water once they were exposed in air for a few days after growth. Such enhanced hydrophobicity in slightly aged 2D MoS_2_ layers has been attributed to a few factors, e.g., accumulation of hydrocarbons on their surface obtained from ambient air^43,45^. More detailed discussion regarding the influence of the surface properties of 2D MoS_2_ layers on the efficacy of water-assisted spontaneous layer separation is presented in the Discussion section.

### Layer-by-layer integration of various 2D TMDs onto unconventional substrates

**Figure 3:**
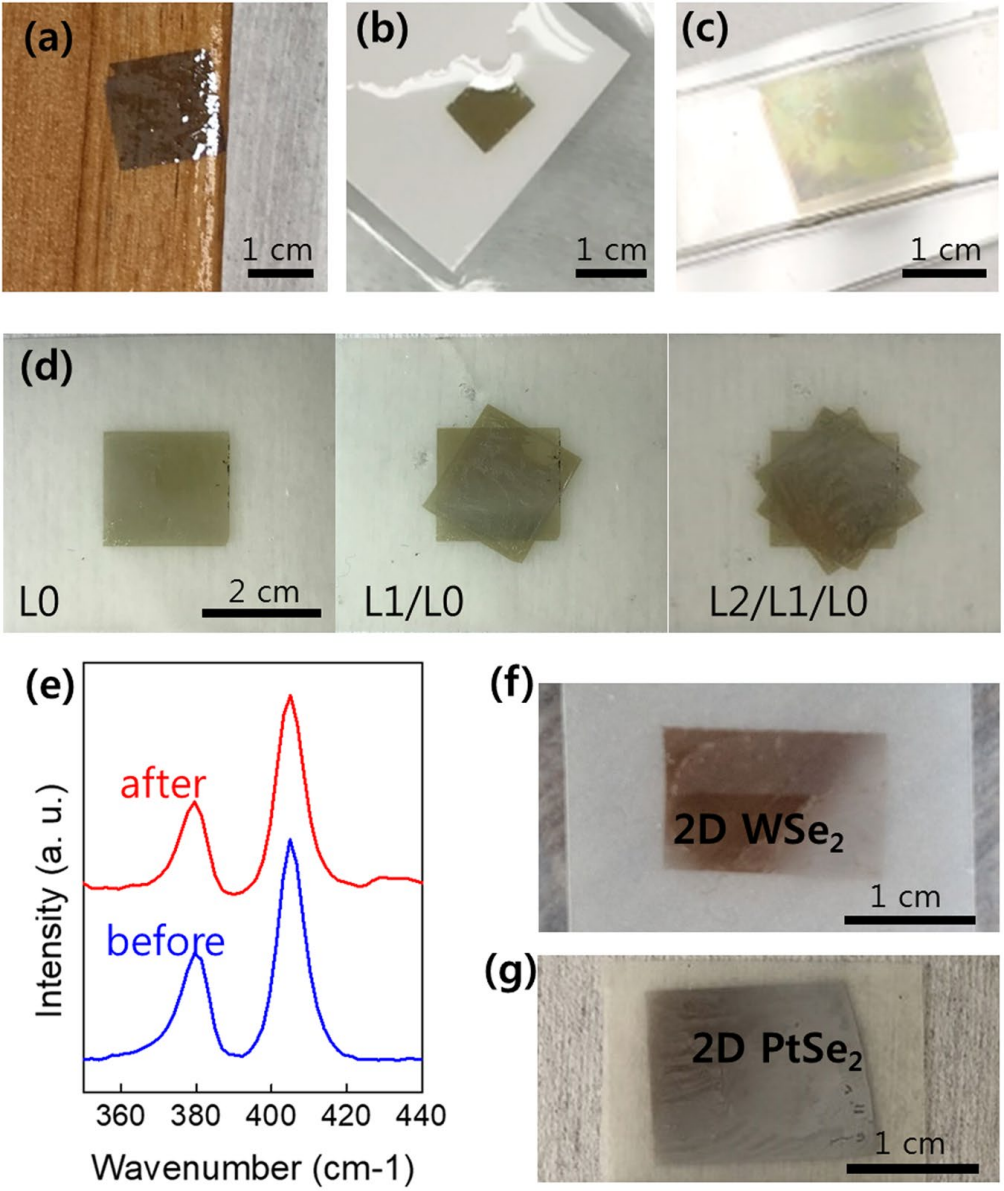
(**a**–**c**) Demonstration of the water-assisted integration of 2D MoS_2_ layers onto a variety of unconventional substrates, including (**a**) wood, (**b**) paper, and (**c**) PDMS. (**d**) Demonstration of the layer-by-layer integration of 2D MoS_2_ layers onto a PET substrate. (**e**) Raman spectra obtained from 2D MoS_2_ layers before and after their water-assisted transfer. (**f**,**g**) Integration of 2D WSe_2_ layers (**f**) and and 2D PtSe_2_ layers (**g**) onto PET substrates.

Figure 3 shows the successful demonstration of the water-assisted integration of 2D TMDs onto a variety of unconventional substrates which would be difficult to achieve otherwise. Figure 3(a–c) shows the images of centimeter-scale 2D MoS_2_ layers integrated on a piece of (a) wood, (b) paper, and (c) cured polydimethylsiloxane (PDMS), respectively. The results highlight the strength and generality of our water-assisted integration approach which is insensitive to the kind of acceptor substrates as the entire process is chemically benign. Figure 3(d) demonstrates the “layer-by-layer” integration of 2D MoS_2_ layers on a piece of polyethylene terephthalate (PET) substrate achieved over an area of ~2 cm^2^. After the initial integration of original 2D MoS_2_ layers (denoted, L_0_), additional layers prepared from the identical substrate were subsequently stacked on them, denoted as (L_1_) and (L_2_), solely via the water-assisted layer transfer. Details for the sequential procedures of this layer-by-layer integration are presented in Supplementary Information, S3. Figure 3(e) compares the Raman spectroscopy profiles of the corresponding sample, L_0_, before (blue) and after (red) its transfer from the original SiO_2_/Si growth substrate. It is evident that the 2D MoS_2_ layers integrated on PET exhibit Raman characteristics highly comparable to those obtained from their as-grown state. The result evidences the well-retained structural and chemical integrity of 2D MoS_2_ layers even integrated on exotic substrates, strongly emphasizing the high reliability, versatility, and cleanness of the water-assisted transfer method. Moreover, we demonstrate the generality of this layer transfer approach by extending it to a variety of 2D TMDs beyond MoS_2_, including 2D tungsten (W) or platinum (P) diselenides (2D WSe_2_ and 2D PtSe_2_). Figure 3(f,g) show the images of centimeter-scale CVD-grown 2D WSe_2_ and 2D PtSe_2_ layers transferred and integrated on PET substrates, respectively.

### Integration of vertically-stacked heterogeneous 2D TMD layers

**Figure 4:**
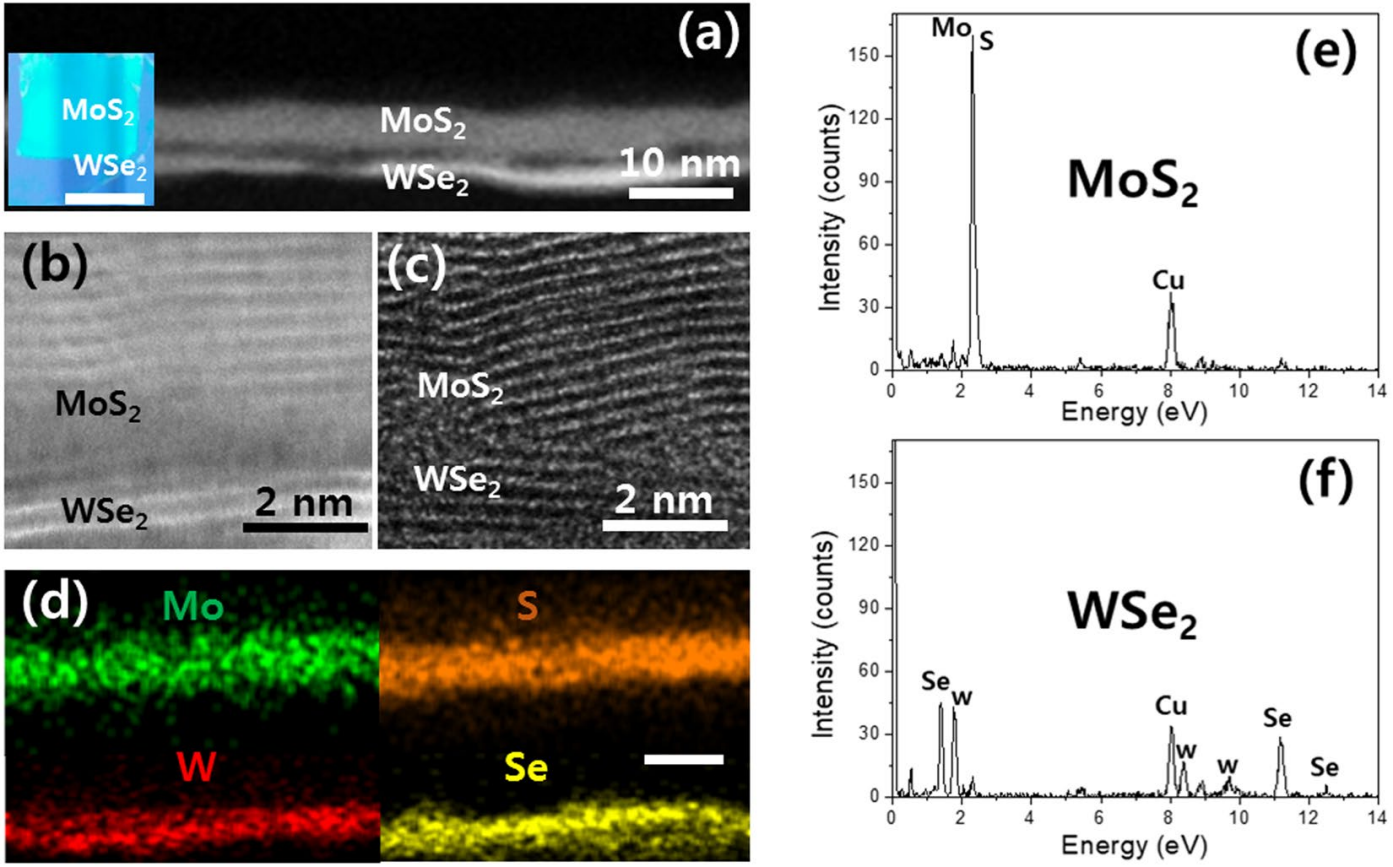
(**a**) Cross-sectional ADF STEM image of a 2D MoS_2_/WSe_2_ hetero vertical-stack. The inset shows the corresponding sample where 2D MoS_2_ layers were integrated on top of 2D WSe_2_ layers. The scale bar is 1 cm. (**b**,**c**) High-resolution ADF-STEM (**b**) and TEM (**c**) images of the corresponding 2D MoS_2_/WSe_2_ hetero-interface. (**d**) STEM-EDS elemental maps to show the spatial distribution of constituent elements in the hetero vertical-stack. The scale bar is 10 nm. (**e**,**f**) EDS profiles obtained from each stack of (**e**) 2D MoS_2_ and (**f**) 2D WSe_2_.

In addition to the demonstration of single-component 2D layer integration, the water-assisted transfer approach can be utilized to fabricate large-area 2D TMD vertical hetero-stacks composed of mixed chemical components, which are difficult to achieve otherwise. Most of the previously developed CVD-grown 2D TMD vertical hetero-stacks share chalcogen anions in each layer, e.g., S in 2D MoS_2_/WS_2_^46–49^ or Se in 2D MoSe_2_/WSe_2_^50–52^ while their lateral sizes are generally limited to nano-to-micrometer dimensions^48–52^. It has been difficult to grow large-area 2D TMD vertical hetero-stacks composed of “mixed” cation/anion components (e.g., 2D MoS_2_/WSe_2_) with conventional CVD approaches. The major limitation stems from that the conventional methods employ the sequential growth of one material (e.g., 2D MoS_2_) on top of the other (e.g., 2D WSe_2_) via step-wise selenization/sulfurization, which often introduces undesired thermal degradation in the pre-grown layers. For example, it has been known that the sequential thermal sulfurization of pre-grown selenides-based 2D TMDs (e.g., MoSe_2_ or WSe_2_) leads to their spontaneous conversion to sulfides-based ones (e.g., MoS_2_ or WS_2_) instead of retaining the original stoichiometry^53,54^. Motivated by this challenge, we explored large-area 2D/2D vertical hetero-stacks of “mixed” cation/anion components, i.e., 2D MoS_2_/WSe_2_, by integrating 2D MoS_2_ layers on top of pre-grown 2D WSe_2_ layers by applying the water-assisted layer transfer. The structural and chemical quality of the 2D MoS_2_/WSe_2_ vertical hetero-stacks was assessed by extensive TEM characterization. The cross-sectional annular dark-field (ADF) scanning TEM (STEM) image in Fig. 4(a) reveals the interfacial morphology of 2D MoS_2_/WSe_2_ vertical hetero-stacks (inset) prepared by integrating 2D MoS_2_ layers on top of pre-grown 2D WSe_2_ layers. Each sample of 2D MoS_2_ layers and 2D WSe_2_ layers was prepared by the CVD sulfurization of Mo (thickness ~2.5 nm) and selenization of W (thickness ~1 nm), respectively. The distinguishable ADF STEM image contrast across the interface indicates the atomic mass (Z-contrast) difference of the constituent materials in each stack region. The enlarged views of the 2D MoS_2_/WSe_2_ hetero-interface are provided in ADF-STEM (Fig. 4(b)) and high-resolution TEM (HRTEM) images (Fig. 4(c)). Both the images clearly reveal that 2D layers are vertically-stacked in a layer-by-layer manner with well-resolved vdW gaps. The brighter image contrast for the bottom stack (WSe_2_) over the top one (MoS_2_) in ADF-STEM reflects the heavier mass of W over Mo. The chemical compositions of the 2D MoS_2_/WSe_2_ vertical hetero-stacks were characterized by energy dispersive X-ray spectroscopy (EDS)-STEM (Fig. 4(d–f)). Figure 4(d) shows EDS elemental map images of the corresponding hetero-interface, revealing the spatial distribution of constituent atomic elements, Mo, W, S, and Se. The images confirm the spatially and chemically localized presence of 2D MoS_2_ and 2D WSe_2_, consistent with the observation in ADF-STEM. Figure 4(e,f) show the EDS spectrum profiles separately collected from each 2D MoS_2_ and 2D WSe_2_ stack, respectively. The results indeed confirm the stoichiometric presence of 2D MoS_2_ and 2D WSe_2_ in each vertical stack, consistent with all other TEM characterization results. It is worth mentioning that the EDS profiles do not exhibit additional peaks from any unintentionally introduced elements excepts for the Cu peaks which are from the TEM grid, further supporting the intrinsic cleanness of our water-assisted layer transfer process.

### Device applications of water-assisted 2D layer transfer and integration

**Figure 5:**
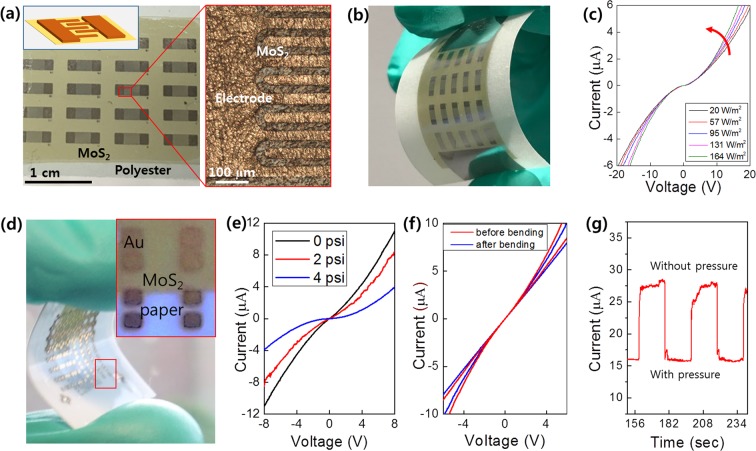
(**a**–**c**) Large-area 2D MoS_2_ layers-based photodetectors integrated on polyester substrates. (**a**) Image of 2D MoS_2_ layers integrated on a flexible polyester substrate with IDEs (schematic in the inset) fabricated on the surface. The zoom-in optical microscopy image (right) shows the array of IDEs (50 μm spacing) on 2D MoS_2_ layers. (**b**) The same device under mechanical bending. (**c**) I–V characteristics obtained at various illumination intensities. (**d**–**g**) Large-area 2D MoS_2_ layers-based pressure sensors integrated on paper substrates. (**d**) Image of a device with metal (gold, Au) contacts under mechanical bending. (**e**) I–V characteristics obtained with varying pressure levels. (**f**) I–V characteristics for a pressure sensing of 3 psi with a device before/after slight mechanical bending. This device is different from the one used for (**e**). (**g**) Time-current characteristics from a pristine unbent device under a periodic pressure application of 5 psi.

We demonstrate the versatility of this water-assisted layer integration method for realizing a variety of large-area 2D MoS_2_ layer devices assembled on unconventional substrates. Specifically, we showcase two examples of 2D MoS_2_-based photodetectors integrated on plastic substrates (Fig. 5(a–c)) and pressure sensors integrated on paper substrates (Fig. 5(d–g)). The photodetectors were fabricated by integrating 2D MoS_2_ layers on polyester substrates followed by depositing an array of interdigitated electrodes (IDEs) on the surface using a shadow mask. Figure 5(a) presents an image of a fabricated 2D MoS_2_ layers/polyester device showing multi-fingered IDEs (zoomed-in image) containing 20 individual device units. Figure 5(b) shows a camera image of the same device under bending, demonstrating its mechanical flexibility. We tested the photo-responsiveness of the device by characterizing its two-terminal current-voltage (I–V) transfer characteristics under an optical microscopy illumination of varying intensity. Figure 5(c) shows that the device exhibits systematically increasing current values with increasing illumination intensity, revealing its excellent photo-sensitivity. The pressure sensor devices were fabricated by integrating large-area 2D MoS_2_ layers on PMMA-coated paper substrates followed by depositing an array of metal electrodes. Figure 5(d) shows an image of a completed device confirming its excellent flexibility under mechanical bending. Figure 5(e) exhibits I–V characteristics with varying pressure levels exerted by the blow of argon (Ar) gas onto the surface of 2D MoS_2_ layers, revealing decreasing current with increasing pressure. Figure 5(f) presents the well-retained pressure sensitivity of a device, which was obtained before and after its slight mechanical bending. Figure 5(g) shows time-current characteristics from a device under the periodic application of Ar pressure, confirming its highly reversible sensitivity.

## Discussion

### Principle for water-assisted spontaneous 2D layer separation

Lastly, we discuss about the fundamental principle behind the facile delamination of 2D layers inside water. We identify followings as two major factors; i.e., crystallographic layer orientation and surface wetting property of 2D MoS_2_ layers.

**Figure 6:**
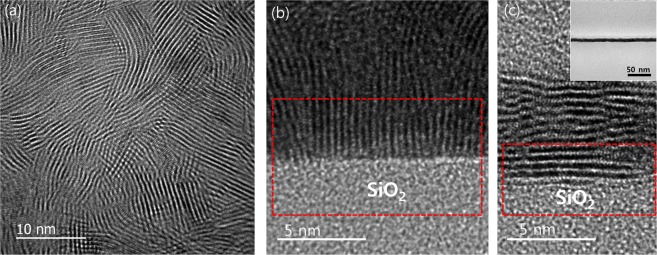
(**a**) Plane-view and (**b**) cross-sectional HRTEM images of vertically-aligned 2D MoS_2_ layers grown on SiO_2_. 2D MoS_2_ layers are rooted in the SiO_2_ surface (red highlight in (**b**)). (**c**) Cross-sectional HRTEM image of horizontally-aligned 2D MoS_2_ layers grown on SiO_2_. 2D basal planes are in contact with the SiO_2_ surface. The inset shows a low magnification TEM image of the corresponding sample.

**Influence of 2D layer orientation**: We have observed that the spontaneous delamination of 2D layers inside water happens with 2D TMDs obtained from the sulfurization of thin metal seeds (typically <3 nm). 2D TMDs obtained in this manner exhibit horizontally-aligned basal planes of well-resolved layers; for example, horizontally-aligned 2D MoS_2_ layers of ~7–8 nm thickness obtained from the sulfurization of ~2.5 nm thick Mo have been confirmed by atomic force microscopy (AFM) and cross-sectional TEM characterization (Supplementary Information, S4). Maintaining the small thickness of metal seeds is critically important to ensure that 2D TMD layers grow in horizontal orientation as identified in our previous studies^32,34^. In this case, weak molecular vdW attraction will be responsible for holding the 2D basal planes of diminished defect density to the SiO_2_ substrate surface, enabling facile water penetration in between them. Meanwhile, we have identified that the CVD sulfurization of thick (typically ~6–7 nm) metal seeds tends to grow 2D TMD layers in vertical alignment. Figure 6(a,b) presents the plane-view and cross-sectional HRTEM images of vertically-aligned 2D MoS_2_ layers prepared with the CVD sulfurization of ~7 nm thick Mo. It is noted that their 2D edge sites are predominantly exposed on the top surface (Fig. 6(a)) while 2D layers are vertically “rooted” in the SiO_2_ growth substrate (Fig. 6(b). This morphology is in sharp contrast to that of horizontally-aligned layers (Fig. 6(c)) and is believed to result from the actual chemical bonding of 2D MoS_2_ edges and SiO_2_ instead of weak vdW attraction. Accordingly, it has been identified that the water-assisted spontaneous separation of vertically-aligned 2D layers is much more difficult in terms of retaining their original shape and dimension on a large area. We have previously identified that 2D TMDs tend to rearrange their layer orientation with increasing physical confinement (increasing metal thickness) in a way to release the accumulating in-plane strain exerted by interconnecting 2D layers^32^. Moreover, we considered the theoretical condition to satisfy the water-assisted spontaneous separation of 2D layers by considering its associated surface energy contributions. The work of adhesion, W, required for the spontaneous 2D layer separation is expressed as ${\rm{W}}={{\rm{\gamma }}}_{{\rm{SW}}}+{{\rm{\gamma }}}_{{\rm{WL}}}-{{\rm{\gamma }}}_{{\rm{SL}}}$, where γ_SW_, γ_WL_, and γ_SL_ are interfacial tension between substrate and water, water and 2D layer, substrate and 2D layer, respectively. In comparing the 2D MoS_2_ layers of horizontal vs. vertical orientation, we note that vertically-aligned 2D MoS_2_ layers exhibit significantly higher adhesion energy with respect to the SiO_2_ surface owing to the exposure of energetically reactive 2D edge sites; i.e., order-of-magnitude higher binding energy of 2D edges with dangling bonds over 2D basal panes with saturated bonds^55,56^. Accordingly, the value of γ_SL_ for vertically-aligned 2D MoS_2_ layers should be much smaller than that for horizontally-aligned ones, which further justifies the observed difficulty with their spontaneous separation inside water. Meanwhile, water can easily penetrate into the vdW gaps exposed in between horizontally-aligned 2D MoS_2_ layers assisted by their large γ_SL_ values. Specific values for these interfacial energy terms are currently unavailable in literature, which makes more precise quantification and elucidation difficult at present.

**Influence of surface wetting property**: As above mentioned, we have observed that 2D MoS_2_ layers aged for a few days after their growth typically exhibit better performance in terms of spontaneous layer separation inside water. In fact, it has been known that the water-wettability of 2D MoS_2_ layers increases with elongated air exposure owing to the lowered surface energy^43,45^, similar to the observation with graphene^57,58^. Accordingly, the surface of 2D MoS_2_ layers becomes more hydrophobic reflected by increasing water contact angle values, which becomes another determining factor for the water-assisted 2D layer separation. The separation efficacy of hydrophobic thin layers inside water has been recently studied^59^, which confirms a presence of strong capillary-induced “peeling” force exerted at their edges interfaced with water. A smaller amount of the capillary force is required to separate the thin layers of higher hydrophobicity while hydrophilic layers tend to get submerged inside water rather than being peeled off^59^. We believe that the identical principle should apply to the water-assisted spontaneous separation of 2D layers presented in this study. Indeed, we have observed that our as-grown 2D MoS_2_ layers of diminished hydrophobicity immersed into water immediately following their growth tend to get submerged without yielding the desired layer separation. This behavior is in sharp contrast to the facile and spontaneous layer separation typically observed with slightly-aged 2D MoS_2_ layers (Supplementary Information, S5).

In summary, we report a novel approach to reliably transfer a variety of 2D TMD layers and heterogeneously integrate them onto virtually arbitrary yet unconventional substrates. The presented method benefits from the water-assisted spontaneous separation of 2D layers, exhibiting distinguishable advantages over conventional approaches in terms of property preservation and size scalability. The intrinsic generality and versatility of this integration method has been verified with 2D TMDs of various kinds as well as their multi-component heterostructures. Moreover, its potential for device applications has been confirmed by demonstrating centimeter-scale 2D MoS_2_-based flexible photodetectors and pressure sensors integrated on exotic substrates, which are difficult to fabricate with any other conventional approaches.

## Methods

### Synthesis of 2D TMDs films

Various 2D TMD layers were grown via the sulfurization or seleneziation of transition metals deposited on SiO_2_/Si substrates using a home-built CVD chamber (Lindberg/Blue M Mini-Mite). High-quality transition metals (e.g., Mo, W, Pt) were deposited on cleaned SiO_2_/Si wafers (typical dimension: ~1 × 3 cm^2^) using an e-beam evaporation system (Thermionics VE-100). The metal-deposited SiO_2_/Si substrates were placed at the center zone of the CVD furnace while alumina boats containing S or Se powders were located at the upstream side. After the furnace was evacuated to the base pressure of ~1 mTorr, high purity Ar gas was flushed for ~10 min to remove any residuals in the chamber. The CVD furnace was gradually ramped up to reach the targeted growth temperatures, i.e., 800 °C for MoS_2_ and WSe_2_, and 400 °C for PtSe_2_, while the S and Se powders were vaporized at ~200 °C. The reaction was maintained for ~45 min under a constant supply of Ar gas at a flow rate of 100 sccm (standard cubic centimeter per minute) followed by natural cooling.

### Structural characterization

TEM characterization was performed using a JEOL ARM200F Cs-corrected TEM at an accelerating voltage of 200 kV. TEM samples were prepared by transferring 2D TMD films from SiO_2_/Si substrates to TEM grids by using water without compromising their intrinsic material quality of as-grown states. Cross-sectional TEM samples (2D MoS_2_ in Fig. 2 and 2D MoS_2_/WSe_2_ vertical-stack in Fig. 4) were prepared by focused ion beam (FIB)-based milling and lift-out techniques. Raman characterization was performed using a Renishaw system with a solid-state laser (spot size: 1 µm) at the excitation wavelength of 532 nm. Optical microscopy images were recorded in an Olympus BX60M optical microscope.

### Device fabrication and characterization

Flexible photodetector devices were fabricating by depositing contact metals through an IDE shadow mask (Ossila) on the pre-integrated 2D MoS_2_ layers on polyester substrates. Photocurrent measurements were performed using a white LED light illumination of controlled intensity and I–V transfer characteristics were recorded by a semiconductor parameter analyzer (HP 4156 A) connected with a home-built probe station. Flexible pressure sensor devices were fabricated by integrating 2D MoS_2_ layers onto PMMA-coated cellulose papers followed by the deposition of top contact metals. Pressure sensing measurements were performed by introducing Ar gas of controlled pressure levels through a quarter-inch vacuum tubing using a flow controller.

## Supplementary information

Supplementary Information S1

Supplementary Information S2

Supplementary Information S3–S5
