# Supplementary material for: Centimeter-scale Green Integration of Layer-by-Layer 2D TMD vdW Heterostructures on Arbitrary Substrates by Water-Assisted Layer Transfer
Source: Sci Rep. 2019 Feb 7;9:1641. doi: 10.1038/s41598-018-37219-w (PMC6367468; doi:10.1038/s41598-018-37219-w)
Supplement: Supplementary file 3 — Supplementary Information S3–S5 [file 41598_2018_37219_MOESM3_ESM.pdf]

Supplementary Information

Centimeter-scale Green Integration of Layer-by-Layer 2D TMD vdW Heterostructures on Arbitrary Substrates by Water-Assisted Layer Transfer

Jung Han Kim,[+,1] *Tae-Jun Ko,*[+,1] *Emmanuel Okogbue,*[1,2] *Sang Sub Han,*[1,3] *Mashiyat Sumaiya Shawkat,*[1,2] *Md Golam Kaium,*[1] *Kyu Hwan Oh,*[3] *Hee-Suk Chung,*[4] *Yeonwoong Jung*[1,2,5,*]

[1]NanoScience Technology Center, University of Central Florida, Orlando, Florida 32826, USA

[2]Department of Electrical and Computer Engineering, University of Central Florida, Orlando, Florida 32816, USA

[3]Department of Material Science and Engineering, Seoul National University, Seoul, 08826, South Korea

[4]Analytical Research Division, Korea Basic Science Institute, Jeonju 54907, South Korea

[5]Department of Materials Science and Engineering, University of Central Florida, Orlando, Florida 32826, USA

Corresponding Authors

*Email: Yeonwoong.Jung@ucf.edu

[+] These authors equally contributed to the work.

Movie S1: Water-assisted 2D layer separation

Movie S2: Water-assisted 2D layer separation

Supplementary Information, S3: Layer-by-layer integration procedure

Supplementary Information, S4: AFM and TEM characterization data.

Supplementary Information, S5: Water immersion of 2D MoS_2 layers in distinct surface states.

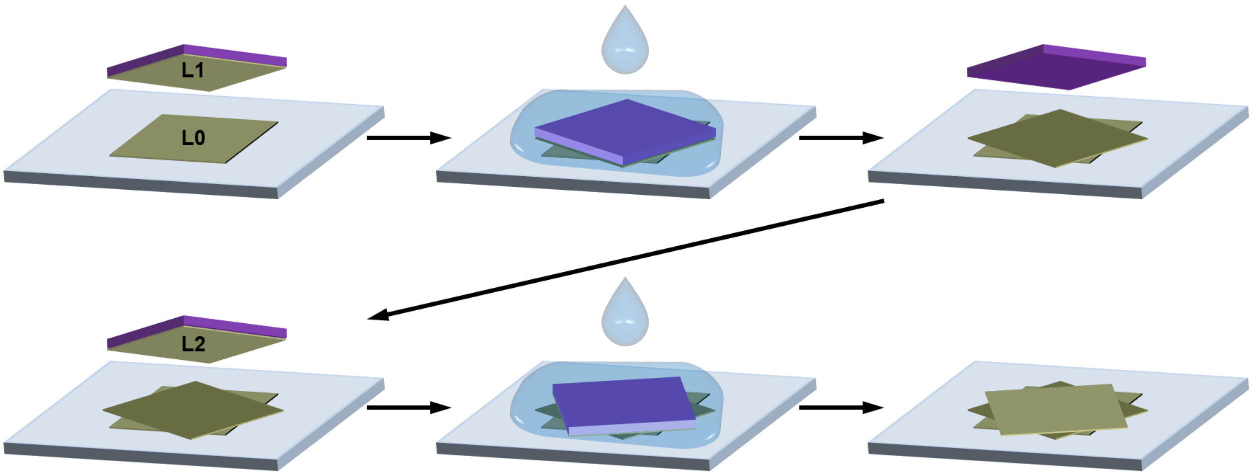

S3. Schematic illustration for the layer-by-layer integration corresponding to Figure 3(d)

The layer-by-layer integration was conducted by the procedure demonstrated in the above Figure. The first stack of 2D MoS_2 layers was separated from the SiO_2 growth substrate and kept floating on the water surface. Then, the secondary substrate was immersed in water and the delaminated 2D MoS_2 layers were transferred on it by mechanical scooping. The integrated 2D MoS_2 layers/substrate was placed on a hot plate and retained at 60℃ for one hour to dry out residual water. For the second stage of 2D layer integration, a sample of 2D MoS_2 layers/SiO_2 substrate was directly placed on top of the first stack of integrated 2D MoS_2 layers in an up-side-down manner. Then, water droplet was directly applied to the sample allowing for sufficient time until the secondary stacks became spontaneously peeled off. The next integration was carried out in the same manner as the previous layer integration.

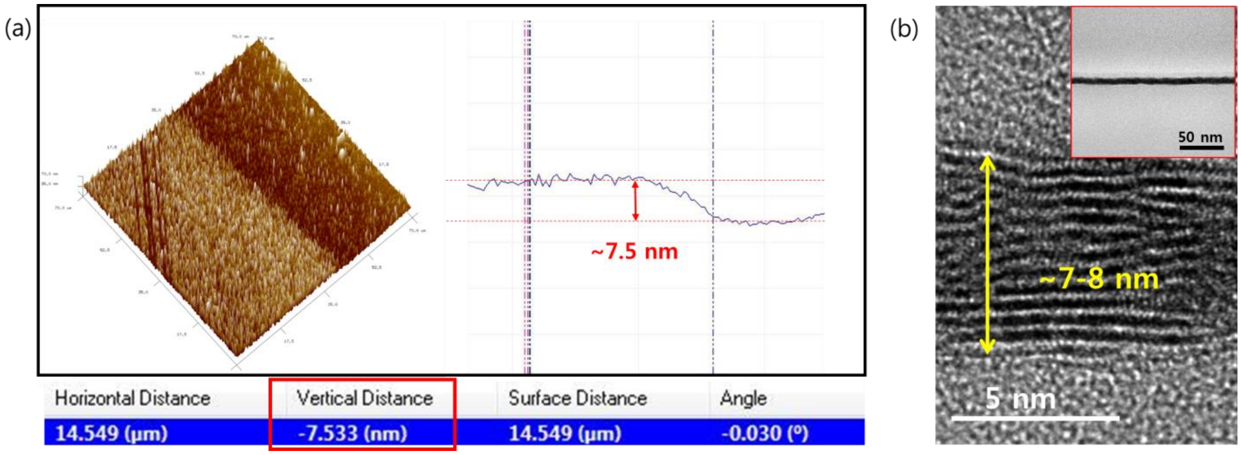

S4. (a) AFM height profile measurement, and (b) cross-sectional TEM image of horizontally-aligned 2D MoS_2 layers of ~7-8 nm thickness.

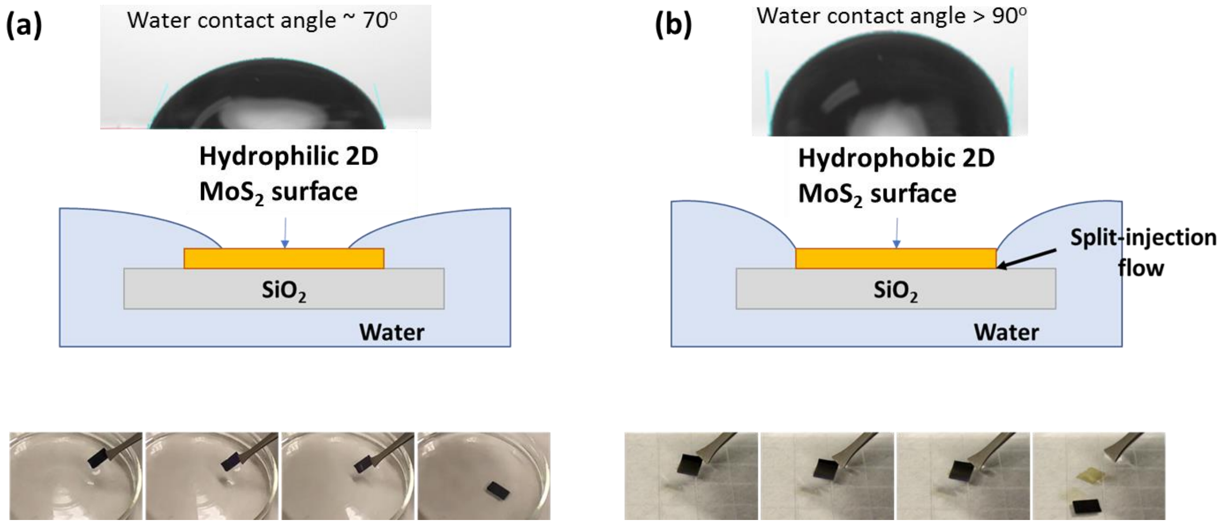

S5. Distinguishable characteristics of 2D MoS_2 layers on SiO_2 substrates upon water immersion when they are in two different surface states. (a) Pristine samples typically get submerged in water. (b) Aged samples yield the facile and spontaneous separation of 2D layers.
